# Comparative analysis of superovulated versus uterine-embryo synchronized recipients for embryo transfer in cynomolgus monkeys (*Macaca fascicularis*)

**DOI:** 10.3389/fvets.2024.1452631

**Published:** 2024-09-13

**Authors:** Dong-Ho Lee, Seung-Bin Yoon, Yu-Jin Jo, Jun Won Mo, Jeongwoo Kwon, Sang Il Lee, Jungkee Kwon, Ji-Su Kim

**Affiliations:** ^1^Primate Resources Center, Korea Research Institute of Bioscience and Biotechnology, Jeongeup, Republic of Korea; ^2^Department of Laboratory Animal Medicine, College of Veterinary Medicine, Jeonbuk National University, Iksan, Republic of Korea

**Keywords:** assisted reproductive technologies, cynomolgus monkey, embryo transfer, pregnancy outcome, recipient

## Abstract

**Introduction:**

Assisted reproductive technologies (ARTs), such as intracytoplasmic sperm injection and embryo transfer, are essential for generating genetically edited monkeys. Despite their importance, ARTs face challenges in recipient selection in terms of time and the number of animals required. The potential of superovulated monkeys, commonly used as oocyte donors, to serve as surrogate mothers, remains underexplored. The study aimed to compare the efficacy of superovulated and uterine-embryo synchronized recipients of embryo transfer in cynomolgus monkeys (*Macaca fascicularis*).

**Methods:**

This study involved 23 cynomolgus monkeys divided into two groups–12 superovulated recipients and 11 synchronized recipients. The evaluation criteria included measuring endometrial thickness on the day of embryo transfer and calculating pregnancy and implantation rates to compare outcomes between groups.

**Results:**

The study found no statistically significant differences in endometrial thickness (superovulated: 4.48 ± 1.36 mm, synchronized: 5.15 ± 1.58 mm), pregnancy rates (superovulated: 30.8%, synchronized: 41.7%), and implantation rates (superovulated: 14.3%, synchronized: 21.9%) between the groups (*p* > 0.05).

**Conclusion:**

The observations indicate that superovulated recipients are as effective as synchronized recipients for embryo transfer in cynomolgus monkeys. This suggests that superovulated recipients can serve as viable options, offering an efficient and practical approach to facilitate the generation of gene-edited models in this species.

## 1 Introduction

Advanced gene-editing technologies such as CRISPR/Cas9 play a pivotal role in generating gene-edited animal models (1), enabling precise modifications in animal embryos. Traditionally, these models have been developed using mice, favoring genetic tractability and cost-effectiveness. However, nonhuman primates (NHPs), which closely mirror humans, offer a more accurate representation of human diseases (2). This feature has been underscored in several studies that have successfully developed gene-edited NHP models (3–6).

Assisted reproductive technologies (ARTs), initially devised to treat infertility in humans, have significantly broadened their applications to include the development of gene-edited animals. These technologies, including *in vitro* fertilization, intracytoplasmic sperm injection (ICSI), and embryo transfer, have been established in NHPs (7–10). However, the development of ARTs has been slow due to financial constraints, limited resources, and the complexity of the procedures.

Cynomolgus monkeys (*Macaca fascicularis*) are preferred for gene-edited NHP models due to their continuous breeding capability, suitable size, and similarities to humans in terms of their reproductive cycles and uterine structure (11). The successful generation of gene-edited cynomolgus monkeys conventionally requires superovulated females for oocyte donation and uterine-embryo synchronized recipients for embryo transfer. This approach can, however, pose challenges because synchronizing the embryo stage with the cycle phase of the recipient candidate is not always straightforward and often necessitates a larger number of female monkeys. Selecting female recipients using a more direct and efficient method for embryo transfer is, therefore, crucial. Very few reports have described the selection of recipients for embryo transfer during the generation of cynomolgus monkeys. Moreover, no previous studies have used donors as recipients for embryo transfer in this species. Research has only reported similar practices in other laboratory animals such as dogs and marmosets (12, 13).

This study, therefore, aimed to compare the efficacy of superovulated and uterine-embryo-synchronized recipients in cynomolgus monkeys for embryo transfer, an essential step in ARTs. By investigating the effects of superovulation on the condition of recipients and pregnancy outcomes, this study sought to enhance the efficiency of developing genetically edited animals using cynomolgus monkeys and contribute to advancing ARTs in this species.

## 2 Materials and methods

### 2.1 Animals

Sexually mature cynomolgus monkeys (88–116 months old) were imported from China by Biomedical Research and housed at the Primate Resources Center (Jeongeup, South Korea). They were individually caged in a room maintained at a temperature of 23 ± 3°C and a humidity of 55 ± 15%. The lighting was regulated on a 12-h light/12-h dark cycle. The monkeys had *ad libitum* access to water and were fed a primate-specific diet supplemented with multivitamins twice daily, with fruits or vegetables provided once daily. Qualified animal caretakers closely monitored all the animals at least twice daily for injuries and illnesses. Additionally, any abnormalities, including signs of pain and unusual behavior, were promptly reported to the veterinarians. Health and medical records were obtained for each animal. All the necessary steps were taken to ensure their well-being and minimize any potential stress or discomfort. All animal procedures performed in this research were in accordance with the ethical standards of the Institutional Animal Care and Use Committee of the Korea Research Institute of Bioscience and Biotechnology (approval numbers: KRIBB-AEC-21306, KRIBB-AEC-24098).

### 2.2 Ovarian stimulation and oocyte recovery

**Figure 1:**
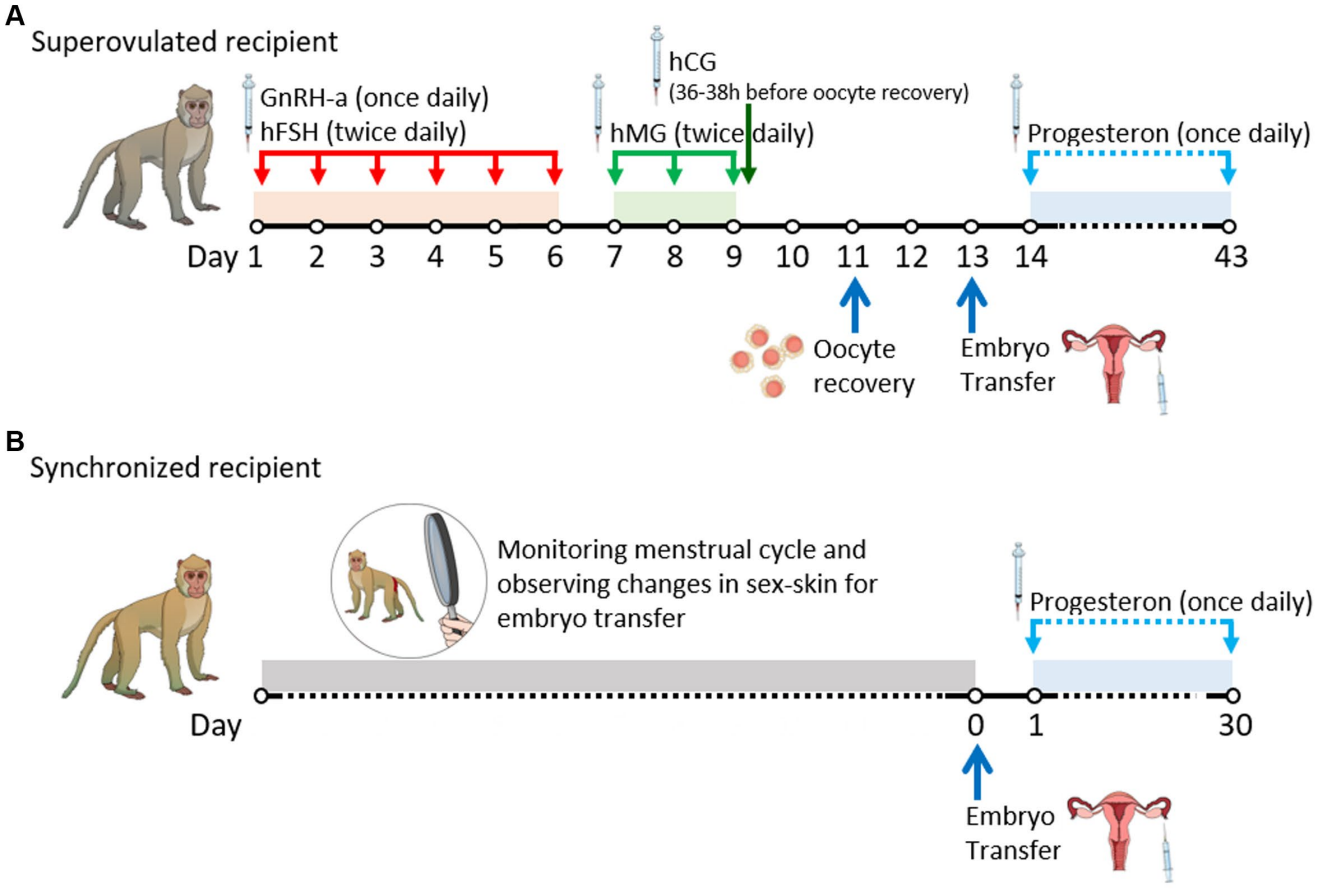
Timeline of experimental procedures by date. **(A)** Schedule of superovulated recipients. **(B)** Schedule of synchronized recipients. GnRH-a, gonadotrophin-releasing hormone antagonist; hCG, human chorionic gonadotrophin; hFSH, human follicle-stimulating hormone; hMG, human menopausal gonadotrophin.

The ovarian stimulation protocol was adapted from previously published studies (14), as illustrated in Figures 1A,B. The regimen included the administration of a gonadotropin-releasing hormone (GnRH) antagonist, ganirelix (Orgalutran Inj, ORGANON, Seoul, Korea), at a dosage of 0.125 mg once daily, and recombinant human follicle-stimulating hormone (hFSH) (Gonal-F Pen, Merck, Serono, Italy) at 37.5 IU twice daily intramuscularly on days 1–6. Human menopausal gonadotropin (IVF-M HP, LG Chem, Cheongju, Korea) was administered at 37.5 IU twice daily intramuscularly on days 7–9. Human chorionic gonadotropin (hCG) (chorionic gonadotropin human, Sigma) was administered intramuscularly at a dose of 1,000 IU 36–38 h before oocyte recovery on day 9. Immediately before oocyte recovery, the developmental status of the follicles was confirmed via ultrasonography (USG), and females with a poor response to stimulation were excluded. During oocyte recovery, the monkeys were anesthetized with an intramuscular dose of 5 mg/kg Zoletil® 50 (Virbac, Carros, France). The ovaries were exposed through an incision in the middle of the lower abdomen, and cumulus-oocyte complexes (COCs) were aspirated using an 18-gage needle attached to a 10.0 mL syringe. The syringe was filled with Tyrode’s albumin lactate pyruvate-4-(2-hydroxyethyl)-1-piperazineethanesulfonic acid (TALP-HEPES) medium, according to a method described by another study (15), supplemented with 4 mg/mL bovine serum albumin (A3311, Sigma, United States) and 5 IU/mL heparin (H3149-25KU, Sigma, USA).

### 2.3 Semen collection

Semen was obtained from male cynomolgus monkeys (71–110 months old) with proven fertility (16) via electrical stimulation and diluted in TALP-HEPES medium supplemented with 5 mg/mL bovine serum albumin. The sperm were then centrifuged at 2,000 rpm for 20 min using a PureSperm 90 gradient (PS90-100, Nidacon, Sweden) to separate the active sperm from the seminal plasma. The supernatant was discarded, and the sperm pellet was further washed by centrifugation at 2,000 rpm for 10 min in PureSperm Wash (PSW-100, Nidacon, Sweden). The top layer of the sperm was collected for use in ICSI.

### 2.4 Intracytoplasmic sperm injection and embryo culture

The COCs were initially rinsed with TALP-HEPES supplemented with 4 mg/mL bovine serum albumin, 5 IU/mL heparin, and 0.2% hyaluronidase (H4272, Sigma-Aldrich) to remove cumulus cells. Oocyte maturation was assessed under an inverted microscope (Leica DMI8; Leica Microsystems, Germany) at magnifications of ×100 or ×200 to identify the germinal vesicle (GV), metaphase I (MI), and metaphase II (MII) stages for analysis. Immature oocytes at the GV and MI stages were then cultured for up to 24 h until they reached the MII stage, in 50 μL drops of mCMRL-1066 medium (11,530,037, GIBCO, United States), supplemented with 10 mM sodium DL-lactate (L7900, Sigma, United States), 25 μg/mL 20% fetal bovine serum (16,000,044, Gibco, United States) and 5 μg/mL PMSG (Pregnant Mare Serum Gonadotropin, Prospec, Israel), 10 ug/ml hCG (CG10, Sigma, United States). The cultures were kept at 37°C in a 5% CO_2_ and 6% O_2_ atmosphere (Heracell 150i, Thermo Fisher Scientific, United States), under embryo-tested mineral oil (M3516, Sigma, United States). MII stage oocytes, either identified or derived from immature oocytes, were injected with monkey spermatozoa for genome editing according to a previously described method (17, 18). For the intracytoplasmic sperm injection (ICSI), spermatozoa were prepared in PureSperm Wash 10 min before the microinjection. A part of the suspended sperm was mixed with 10% polyvinylpyrrolidone. The zona pellucida of oocytes in injection media was covered with mineral oil and penetrated by several piezo pulses. The oolemma was punctured by the application of 1–2 piezo pulses, with the pipette tips reaching the opposite side of the oocyte cortex and the oolemma stretched without being broken. The sperm head was injected into the oocyte cytoplasm with a minimum amount of medium. Injected oocytes were incubated for at least 10 min in micromanipulation medium (TALP-HEPES) for stabilization. The oocytes were then transferred into mCMRL medium containing 0.4% BSA (A3311, Sigma, United States) and further cultured under embryo-tested mineral oil at 37°C in an atmosphere of 5% CO_2_ and 6% O_2_ for 48 h prior to embryo transfer.

### 2.5 Recipient selection

Oocyte donors also served as recipients in the superovulated group. In the synchronized group, recipient selection was based on monitoring the regular menstrual cycle and observing changes in sex-skin color and swelling (Supplementary Figure S1), which typically occur during ovulation between days 13 and 19 after menstruation. These observations established a 6–9 day window period for embryo transfer (19). Additionally, only those with a uterus presenting normal echo, as verified by USG before embryo transfer, were selected as embryo recipients in both groups.

### 2.6 Embryo transfer

Embryo transfer was performed 2 days after oocyte recovery, with the monkeys under anesthesia which was administered via an intramuscular dose of 5 mg/kg Zoletil® 50 (Virbac, Carros, France). Only the embryos that reached the four-cell stage were selected. Using a microglass capillary, 1 to 2 μL of BSA-free mCMRL medium containing the selected embryos were carefully picked up from the culture dishes. The embryos were surgically transferred to the oviduct via the infundibulum. In the standard procedure, two to three embryos were deposited in the oviduct. Luteal phase support was provided through daily intramuscular injections of progesterone (Taiyu Progesterone; Taiyu Chemical & Pharm, Taiwan) at a dosage of 3.5 mg, commencing the day after embryo transfer and continuing until ultrasonographic confirmation of pregnancy at 30 days.

### 2.7 Abdominal ultrasonography

USG was employed to assess ovarian conditions in oocyte donor monkeys before oocyte recovery and to evaluate uterine conditions in recipient monkeys before embryo transfer. Specifically, endometrial thickness in recipient monkeys was measured in the transverse plane at the point of the greatest uterine diameter. Pregnancy was diagnosed on day 30 following the transfer, confirming the presence of a yolk sac and embryonic cardiac motion using USG (20) (Supplementary Figure S2; Supplementary Video S1). The procedure was conducted under anesthesia induced by 10 mg/kg ketamine (Yuhan Ketamin 50 Inj., Yuhan Corporation, South Korea) by an experienced veterinarian using a high-resolution ultrasound device (LOGIQ e, GE Healthcare Technologies, Inc., Chicago, IL, United States) equipped with a 12.0 MHz probe.

### 2.8 Statistical analysis

All the data analyses were performed using the GraphPad Prism 8 software (GraphPad Software, LLC). Comparisons between groups for continuous variables were performed using the Student’s t-test. Pregnancy and implantation rates were compared between groups using Fisher’s exact test. The data were presented as mean ± standard deviation (SD). Differences were considered statistically significant at a *p* value of less than 0.05.

## 3 Results

### 3.1 Comparative characteristics of the superovulated vs. synchronized group

Table 1 provides a comparative overview of the characteristics of the superovulated and synchronized recipients in this study. The superovulated group had an average age of 98.4 ± 7.7 months, closely aligning with the average age of the synchronized group of 97.8 ± 9.0 months. The body weight for the superovulated group averaged 3.66 ± 0.45 kg, slightly less than the synchronized group at 3.92 ± 0.59 kg. There was no statistically significant difference in age and body weight between the two groups, as confirmed by the Student’s t-test (*p* > 0.05).

**Table 1:** Comparative characteristics of superovulated vs. synchronized recipients.

	Superovulated group (*n* = 13)	Synchronized group (*n* = 12)
1. Age (month)	98.4 ± 7.7	97.8 ± 9.0
2. Body weight (kg)	3.66 ± 0.45	3.92 ± 0.59
3. *Ultrasonographic findings		
3.1 Ovary diameter (mm)		
3.1.1 Length	19.62 ± 5.32	Not applicable
3.1.2 Width	10.83 ± 2.73	Not applicable
3.2 Endometrial thickness (mm)	4.48 ± 1.36	5.15 ± 1.58

*Ovary size was measured before oocyte recovery and endometrial thickness was measured before embryo transfer. Data are presented as the means ± standard deviation. No significant differences were detected within rows using the Student’s *t*-test (*p* > 0.05).

**Figure 2:**
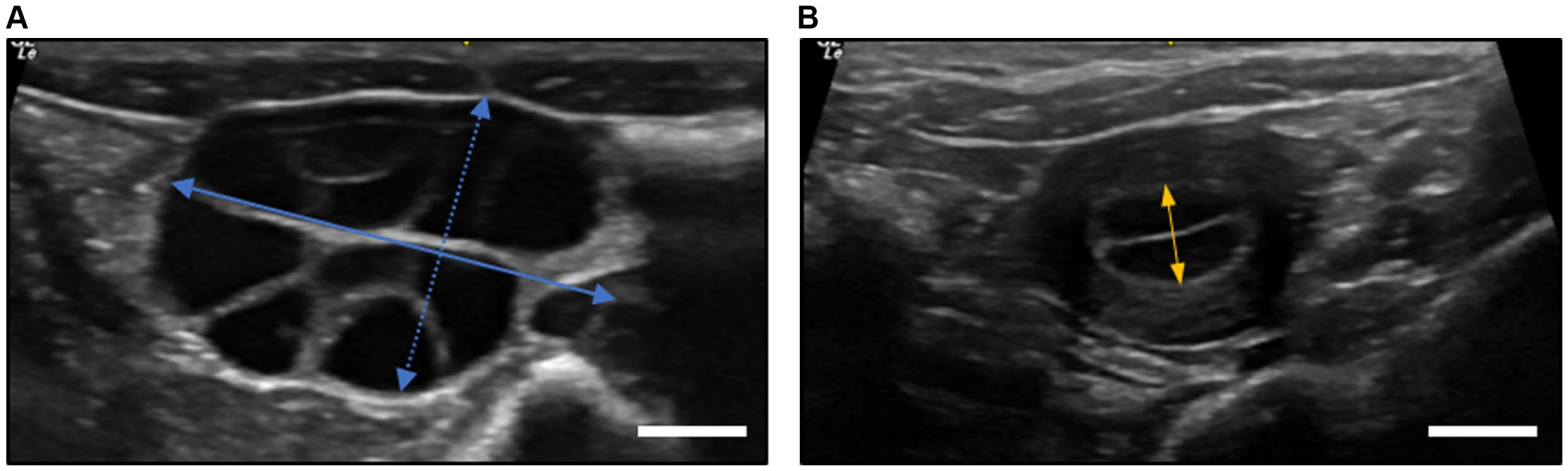
Ultrasonographic measurements of ovary diameter and endometrial thickness in cynomolgus monkeys. **(A)** Ovary diameter after superovulation procedures at oocyte recovery. The length of the ovary was defined as the longest axis (straight blue line), while the width was measured perpendicular to the length at its widest point (dotted blue line). **(B)** The endometrial thickness of recipients at embryo transfer. The measurement was taken at the thickest part of the endometrium, typically in the transverse plane of the uterus (straight orange line). All measurements were taken using a digital caliper in ultrasound device software with an accuracy of ±0.01 mm. The scale bar is 5.0 mm.

The ovary size was measured before oocyte recovery and endometrial thickness was measured before embryo transfer using USG (Figures 2A,B). The ovary size in the superovulated group was measured before oocyte recovery, yielding dimensions of 19.62 ± 5.32 mm in length and 10.83 ± 2.73 mm in width. The ovary size in the synchronized group was not assessed. In terms of the endometrial thickness before embryo transfer, the superovulated group presented a slightly thinner endometrium at 4.48 ± 1.36 mm compared to 5.15 ± 1.58 mm in the synchronized group. However, these differences were not statistically significant, as determined by the Student’s t-test (*p* > 0.05).

### 3.2 Pregnancy outcomes following embryo transfer in two groups

The data on pregnancy and implantation rates are summarized in Table 2. In the superovulated group, 35 embryos were transferred to 13 recipients, resulting in four pregnancies (30.8%), including one twin pregnancy. The remaining participants had singletons. In the synchronized group, 32 embryos were transferred to 12 recipients, leading to five pregnancies (41.7%), one of which was a triplet pregnancy. The implantation rates were 14.3% (five of 35 transferred embryos) in the superovulated group and 24.1% (seven of 32 transferred embryos) in the synchronized group. Although the pregnancy and implantation rates were higher in the synchronized group, these differences were not statistically significant (*p* > 0.05). Additionally, direct observations after embryo transfer revealed skin suture dehiscence in two cases, one in each group, which was attributed to the actions of the monkeys; however, no major infections, incision abnormalities, or other significant complications were observed.

**Table 2:** Pregnancy and implantation rates following embryo transfer in two groups.

Group	Pregnancy rate	Implantation rate
Superovulated recipients	30.8% (4/13, 1 twin)	14.3% (5/35)
Synchronized recipients	41.7% (5/12, 1 triplet)	24.1% (7/32)

No significant differences were detected within the columns using the Fisher’s exact test (*p* > 0.05).

## 4 Discussion

### 4.1 Principal findings of the study

The observations suggest that in embryo transfer, superovulated recipients are as effective as synchronized recipients and could be considered a preferable option because of their comparable pregnancy outcomes in cynomolgus monkeys.

### 4.2 Limitations of previous studies

The superovulated oocyte donor is commonly the recipient in human ARTs; however, its application is limited in NHPs. In NHPs, surrogate recipients have primarily been used to increase the number of offspring produced for research purposes and avoid complications associated with transferring embryos back into the oocyte donor during the stimulation cycle. To date, numerous cynomolgus monkey offspring have been produced using ARTs with and without gene editing methods. To the best of our knowledge, no reports have documented the use of superovulated recipients; instead, most studies have employed embryo-uterus-synchronized recipients (Table 3). Research has predominantly focused on selecting the most suitable synchronized recipients because of the widely recognized importance of aligning the developmental stage of embryos with the uterine conditions of the recipients, which is essential for successful embryo transfer (8, 21).

**Table 3:** Previous studies on recipient selection and pregnancy outcomes in cynomolgus monkeys through ARTs.

Previous studies	Year	ARTs	Gene-editing method	Recipient selection	Pregnancy rate (%)	Implantation rate (%)
Sun et al. (8)	2008	IVF, ICSI	None used	Synchronized recipients	27.6	13.6
Liu et al. (5)	2014	IVF	TALEN	Synchronized recipients	31.6	14.8
Niu et al. (3)	2014	ICSI	CRISPR/Cas9	Synchronized recipients	34.5	22.9
Wan et al. (4)	2015	ICSI	CRISPR/Cas9	Synchronized recipients	30.8	21.0
Seita et al. (47)	2016	ICSI	Lentivirus-mediated gene transfer	Not synchronized recipients	66.7	60.0
Ke et al. (48)	2016	ICSI	TALEN	N.A.	33.3	7.7
Zhao et al. (49)	2017	ICSI	CRISPR/Cas9	N.A.	8.1	2.6
Chen et al. (50)	2017	ICSI	TALEN	Synchronized recipients	34.1	13.0
Zhang et al. (51)	2018	ICSI	CRISPR/Cas9	Synchronized recipients	33.3	8.3
Cui et al. (52)	2018	ICSI	CRISPR/Cas9	Synchronized recipients	30.0	13.3
Zhou et al. (53)	2019	ICSI	CRISPR/Cas9	Synchronized recipients	46.2	18.0
Qiu et al. (54)	2019	ICSI	CRISPR/Cas9	Synchronized recipients	32.3	9.1
Tsukiyama et al. (55)	2019	ICSI	CRISPR/Cas9	Not synchronized recipients	33.7	N.A
Huang et al. (19)	2020	ICSI	None used	Synchronized recipients	25.0	27.3
Schmidt et al. (56)	2020	ICSI	CRISPR/Cas9	Synchronized or not synchronized recipients	0.0	0.0
Wang et al. (57)	2020	ICSI	CBE	Synchronized recipients	54.5	19.5
Chen et al. (58)	2021	ICSI	CRISPR/Cas9	Synchronized recipients	24.0	13.7
Li et al. (59)	2024	FPNT	None used	Synchronized recipients	20.0	11.4

Pregnancy rate = number of pregnant recipients/number of embryo-transferred recipients × 100; implantation rate = number of embryos implanted/number of embryos transferred × 100. CBE, cytosine base editing; CRISPR/Cas9, clustered regularly interspaced short palindromic repeats and CRISPR-associated protein 9; FPNT, female pronucleus transfer; ICSI, intracytoplasmic sperm injection; IVF, in vitro fertilization; N.A., not available; TALEN, transcription activator-like effector nuclease.

### 4.3 Challenges in identifying uterine-embryo synchronized recipients

Identifying uterine embryo-synchronized recipients in cynomolgus monkeys can be challenging. In humans, oocyte donors often serve as recipients and synchronous embryo transfer has been associated with high pregnancy rates (22). Surrogates other than oocyte donors are usually employed in cynomolgus monkeys. The process of identifying a synchronized recipient typically involves confirming synchronization through various methods such as assessing estradiol levels, monitoring menses and sex-skin changes, and observing new stigma or new corpus luteum in the ovaries via USG or laparoscopy, either separately or in combination.

Hormone assays that detect serum estradiol levels are commonly used, with embryo transfer typically occurring 1–3 days after a peak in estradiol levels (8). However, this process requires daily blood collection over a long period to monitor estradiol levels, as the peak can occur anywhere from seven to 20 days after menstruation (19). This can cause stress in monkeys and complicate the synchronization of embryonic development with the recipient’s uterine condition.

Predicting the ovulation date by monitoring the menstrual cycle and sex-skin changes may be straightforward. The menstrual cycle of cynomolgus monkeys is approximately 29 days, with ovulation occurring approximately 11–14 days after the onset of menstruation (23, 24). Sexual swelling and reddening are highly accurate indicators of ovulation timing (25). Nevertheless, monitoring a regular menstrual cycle is time-consuming, and not all female’s exhibit changes in sex-skin (26).

The technique of detecting ovulation through the observation of a new stigma or corpus luteum using USG or laparoscopy is highly accurate for confirming synchronization. This method, however, requires specialized equipment and skilled personnel and may not always detect ovulation points during a normal menstrual cycle (19).

Selecting a synchronized recipient, therefore, requires significant time and resources as well as several female monkeys. This increases the complexity and ethical challenges involved in effective embryo transfer in NHP studies.

### 4.4 Analysis of endometrial thickness in superovulated and synchronized recipients

The endometrium of cynomolgus monkeys undergoes significant changes during the menstrual cycle, which are primarily driven by ovarian hormones and their receptors (26, 27).

The endometrium typically expands during the follicular phase and reaches its peak immediately after ovulation (28), and is considered a critical aspect of uterine receptivity. Uterine receptivity is crucial for embryo transfer, not only in humans but also in NHPs (29, 30).

In humans, studies on the effect of endometrial thickness have shown varied outcomes, with some studies suggesting a more favorable outcome for pregnancies with an endometrial thickness of at least 10 mm and negative outcomes with thicknesses below 6 mm (31, 32) while others have reported successful pregnancies with thicknesses as low as 4 mm (33). Interestingly, additional research has indicated that endometrial thickness may not be directly related to pregnancy outcomes (34).

The comparison of endometrial thicknesses measured using ultrasound revealed no statistically significant differences between the superovulated and synchronized groups in this study. Furthermore, the observations presented no significant differences compared to those of a previous study, which reported an endometrial thickness of 5.7 mm (35). These findings suggest that the endometrial changes induced by superovulation are comparable to those occurring during the natural menstrual cycle and do not adversely affect the endometrial thickness, implying that superovulated recipients have a uterine receptivity similar to that of synchronized recipients.

For additional analysis, the results of this study were categorized into pregnant and non-pregnant recipients, with endometrial thicknesses measured at 4.89 ± 0.71 mm and 4.75 ± 1.79 mm, respectively. No significant differences were observed between the two groups (*p* > 0.05). The results suggest that endometrial thickness does not significantly affect pregnancy outcomes in cynomolgus monkeys, because endometrial thickness is often considered a factor in successful implantation and pregnancy.

### 4.5 The effect of superovulation on the pregnancy outcomes

In human ARTs, the impact of controlled ovarian hyperstimulation—the use of hormonal medications to stimulate the ovaries to produce multiple follicles, similar to superovulation in this study—on pregnancy and implantation rates has been extensively studied. Several studies have indicated that hyperstimulation does not negatively affect endometrial receptivity or pregnancy outcomes (36–39); however, others have highlighted the potential detrimental effects on the outcomes of assisted reproduction (40–43). Laboratory animal studies involving rats and mice have demonstrated mixed results in terms of the effects of ovarian hyperstimulation. Research on rats suggests that hyperstimulation can maintain normal uterine receptivity (44), while findings from mouse studies indicate potential negative impacts on implantation due to endometrial alterations (45). Despite this controversy, ovarian hyperstimulation is a critical component of fertility treatments and the generation of mutant animals, enhancing both the number of oocytes and the quality of embryos available for fertilization and subsequent development.

In the current study, no significant differences were observed in the pregnancy and implantation rates between the superovulated and synchronized recipient groups. It is believed that a superovulation protocol can effectively optimize the conditions for both follicular development and endometrial preparation by forcefully controlling the menstrual cycle. Furthermore, the administration of progesterone after embryo transfer, which is commonly used in human fertility treatments to aid embryo implantation and maintain pregnancy (46), is considered to have similar beneficial effects.

This study additionally achieved moderate success rates for pregnancy and implantation, comparable to those reported in other studies (Table 3). These findings support the effective use of superovulated recipients as synchronized recipients for embryo transfer in cynomolgus monkeys.

### 4.6 Strengths and weaknesses of the study

This innovative study confirmed that superovulated monkeys, traditionally used only as oocyte donors, can also serve as surrogate mothers. This finding is beneficial in terms of the time, cost, and reduction in the number of animals needed, as well as alleviating the cumbersome process associated with selecting surrogate recipients. The meticulous division and control of the two groups enhanced the reliability of the findings, suggesting that both methods were equally effective. Moreover, the study included measurements of endometrial thickness, which not only enhanced the understanding of the effects of superovulation but also enabled comparisons of endometrial thickness between pregnant and non-pregnant monkeys. This detail is particularly relevant as it may influence clinical approaches to reproductive technologies. This study, therefore, makes a practical contribution by identifying superovulated recipients as viable and efficient alternatives for generating genetically edited models and broadening the knowledge base of ARTs in cynomolgus monkeys.

This study was, however, limited by its small sample size, which may have restricted the generalizability of the results. The slightly higher pregnancy and implantation rates observed in the synchronized group suggest a trend that may become more apparent with larger sample sizes. Focusing predominantly on short-term outcomes additionally limits a comprehensive understanding of the long-term implications of these ART methods, such as complications of repeated surgery, pregnancy maintenance, and birth rates.

### 4.7 Unanswered questions and proposals for future studies

In future studies, it will be necessary to confirm these findings in superovulated recipients with larger sample sizes, slightly different superovulation protocols, and long-term outcomes to ensure the reliability and applicability of the results. Additionally, exploring biological mechanisms such as hormonal profiles, endometrial gene expression, and the uterine microenvironment in superovulated recipients will provide deeper insights into the underlying processes. Employing refined surgical methods such as laparoscopy can enhance animal welfare by reducing stress and increasing the safety of procedures. Ultimately, these studies will improve the overall efficacy and safety of ARTs for cynomolgus monkeys.

## 5 Conclusion

**Figure 3:**
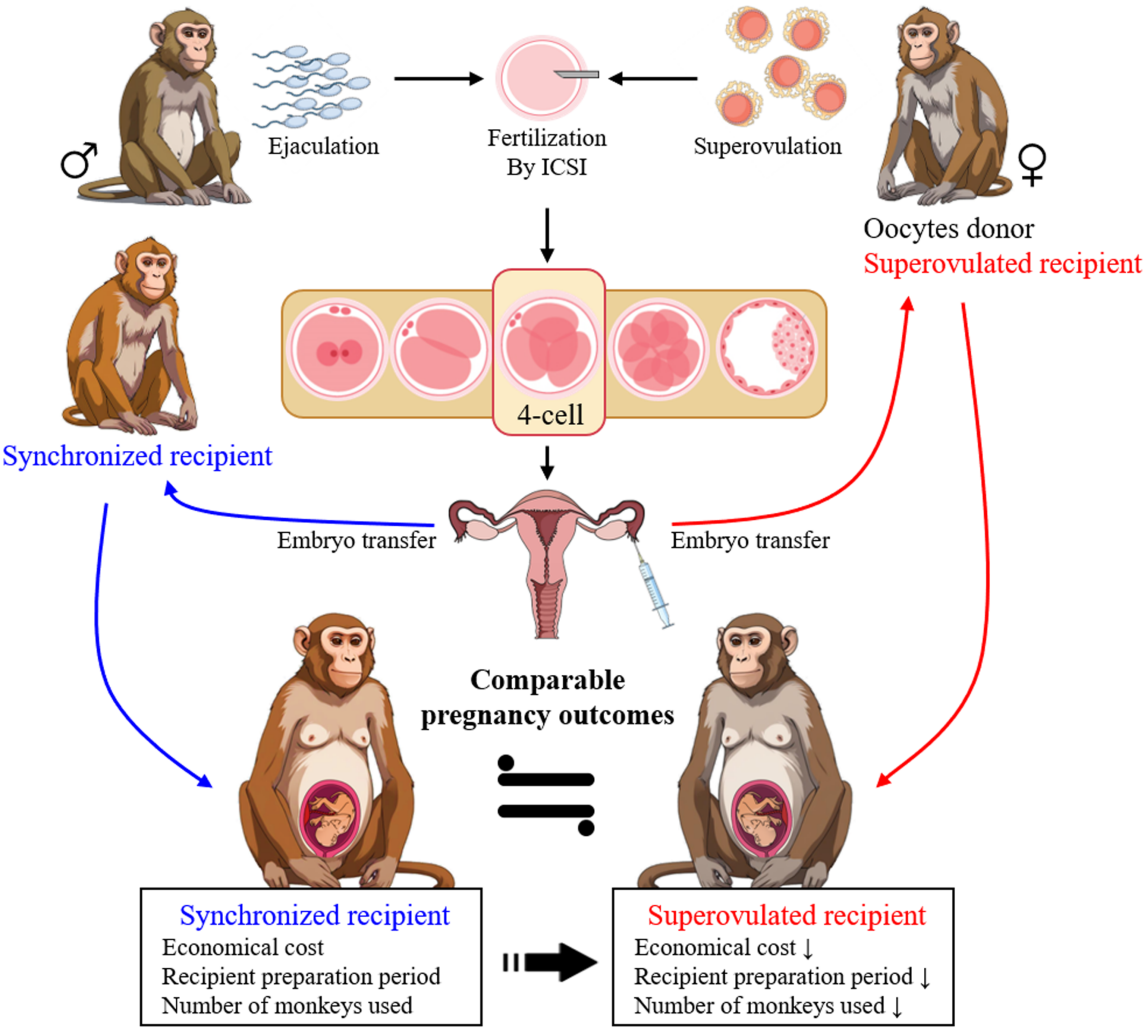
The highlight of superovulated recipients as effective surrogates compared to synchronized recipients in embryo transfer for generating gene-edited monkeys. The selection of superovulated recipients enhances efficiency and the practical application of ARTs in this species.

ARTs are vital for producing mutant monkeys; they, however, often encounter challenges in recipient selection. To the best of our knowledge, this is the first study to compare superovulated and uterine-embryo synchronized recipients in cynomolgus monkeys. The observations from this study highlighted that superovulated recipients, who are also oocyte donors, effectively serve as surrogates (Figure 3). This approach not only simplifies recipient selection and reduces the number of animals needed but also enhances the practical application of ARTs, facilitating the creation of gene-edited models in this species.

## Data Availability

The raw data supporting the conclusions of this article will be made available by the authors, without undue reservation.
