# Supplementary material for: Comparative analysis of superovulated versus uterine-embryo synchronized recipients for embryo transfer in cynomolgus monkeys (Macaca fascicularis)
Source: Front Vet Sci. 2024 Sep 13;11:1452631. doi: 10.3389/fvets.2024.1452631 (PMC11427438; doi:10.3389/fvets.2024.1452631)
Supplement: Supplementary file 2 [file Data_Sheet_1.docx]

***Supplementary Material***

**Supplementary Figure S1**

Synchronized recipient selection based on observing changes in sex-skin color and swelling. **(**A) Characteristics of a selected recipient in the ovulatory stage. The vaginal area is swollen and reddened, with subcutaneous groin forming two sac-like protrusions. (B) Characteristics of an unselected recipient. The turgescent area is obviously narrowed, and the color is less red.


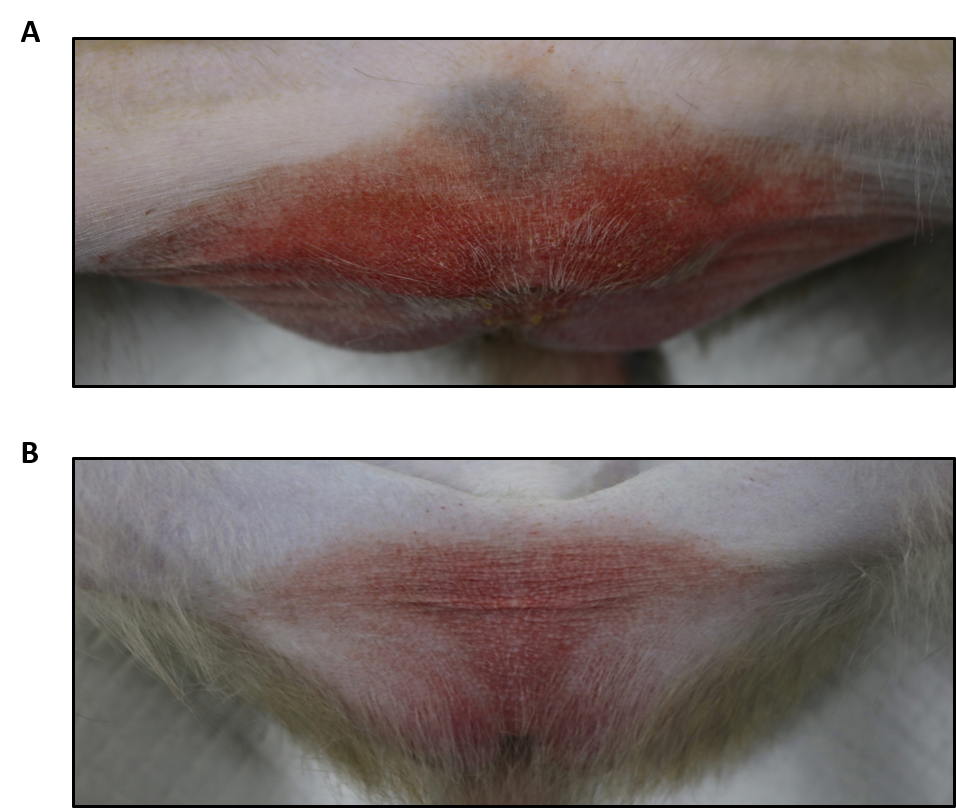


**Supplementary Figure S2**

Ultrasonographic image of a pregnant cynomolgus monkey uterus on day 30 following embryo transfer. The yolk sac (indicated by a yellow arrow) and the embryo with a beating heart (indicated by a blue arrow) are identified within the gestational sac.


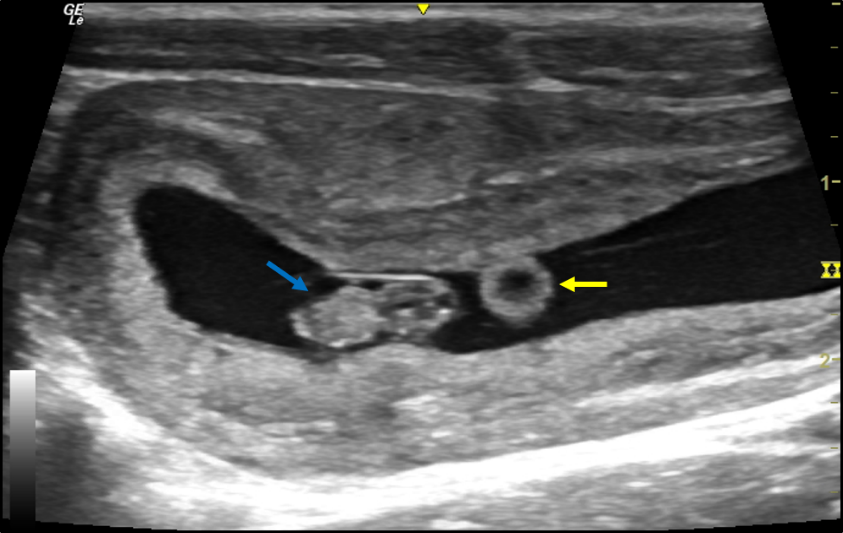


**Supplementary Video S1**

Ultrasonographic video of a pregnant cynomolgus monkey uterus on day 30 following embryo transfer. The video shows a lateral view of the embryo with visible cardiac motion.
